# The Feasibility of Artificial Intelligence and Raman Spectroscopy for Determining the Authenticity of Minced Meat

**DOI:** 10.3390/foods14173084

**Published:** 2025-09-02

**Authors:** Aleksandar Nedeljkovic, Aristide Maggiolino, Gabriele Rocchetti, Weizheng Sun, Volker Heinz, Ivana D. Tomasevic, Vesna Djordjevic, Igor Tomasevic

**Affiliations:** 1 Department of Animal Source Food Technology, Faculty of Agriculture, University of Belgrade, Nemanjina 6, 11080 Belgrade, Serbia; 2 Department of Veterinary Medicine, University of Bari Aldo Moro, Strada Provinciale per Casamassima Km 3, 70010 Valenzano, Italy; aristide.maggiolino@uniba.it; 3 Department of Animal Science, Food and Nutrition, Università Cattolica del Sacro Cuore, Via Emilia Parmense 84, 29122 Piacenza, Italy; gabriele.rocchetti@unicatt.it; 4 School of Food Science and Engineering, South China University of Technology, Guangzhou 510641, China; fewzhsun@scut.edu.cn; 5 DIL German Institute of Food Technologies e.V., Prof.-von-Klitzing-Str. 7, 49610 Quakenbrück, Germany; v.heinz@dil-ev.de; 6 Institute of Meat Hygiene and Technology, Kaćanskog 13, 11000 Belgrade, Serbia; ivana.tomasevic@inmes.rs (I.D.T.);

**Keywords:** adulteration, machine learning, Support Vector Machines, Artificial Neural Network, Random Forest, rapid analysis, chemometrics

## Abstract

Food fraud in meat products presents serious economic and public health challenges, underscoring the need for rapid and reliable detection methods. This study investigates the potential of Raman spectroscopy combined with machine learning to accurately discriminate between pure and mixed minced meat preparations. We evaluated three classification algorithms: Support Vector Machines (SVMs), Artificial Neural Networks (ANNs), and Random Forests (RFs). Raman spectra were collected from 19 distinct samples consisting of different ratios of pork, beef, and lamb minced meat. Our findings suggest that homogenization markedly enhances spectral consistency and classification accuracy. In the pure meat samples case, all three models (SVM, ANN, and RF) achieved notable increases in classification accuracies (from 0.50–0.70 to above 0.85), a dramatic improvement over unhomogenized samples. In more complex homogenized mixtures, SVM delivered the highest performance, achieving an accuracy of up to 0.88 for 50:50 mixtures and 0.86 for multi-ratio samples, often outperforming both ANN and RF. While the underlying interpretation of the classification models remains complex, the findings consistently underscore the critical role of homogenization on model performance. This work demonstrates the robust potential of the Raman spectroscopy-coupled machine learning approach for the rapid and accurate identification of minced meat species.

## 1. Introduction

The global meat industry faces persistent challenges related to food fraud, mislabeling, and economically motivated adulteration [1]. Minced meat products are particularly susceptible to such fraudulent activities due to the loss of distinct morphological characteristics during processing, which makes visual identification of adulterants extremely difficult [2]. Comprehensive reviews [3,4,5,6] have highlighted that meat adulteration, whether intentional or unintentional, significantly infringes upon consumer rights and poses public health risks. There is therefore an urgent and ongoing need for rapid, reliable, and effective analytical methods for identifying meat species and detecting adulteration.

Traditional methods for meat quality evaluation have often relied on destructive, time-consuming, and chemical-dependent protocols. While highly accurate molecular biology techniques like Polymerase Chain Reaction (PCR) and Enzyme-Linked Immunosorbent Assay (ELISA) offer high specificity and sensitivity, their requirement for expensive equipment, technical expertise, and lengthy procedures limits their applicability for high-throughput screening or on-site quality control [4,7]. In response to these limitations, modern methodologies, such as spectroscopy-based technologies, are emerging as promising alternative tools. Raman spectroscopy, in particular, is well suited for this challenge due to its ability to provide a unique molecular fingerprint of a sample [8], its rapid data acquisition, and low interference from water in complex biological matrices [7].

The versatility of Raman spectroscopy has been demonstrated in a range of agri-food applications. Past work has used the method for the quantitative determination of fatty acids in pork [9], species differentiation in beef and horsemeat [10], and the detection of adulteration in dairy products [11,12]. While these studies show promise, the high-dimensional and often complex nature of Raman spectral data requires advanced data processing and interpretation. Machine learning algorithms, including Artificial Neural Networks (ANNs), Random Forests (RFs), and Support Vector Machines (SVMs), offer powerful solutions for building robust predictive models from such datasets [6,7]. Although previous studies have combined machine learning with other spectroscopic methods for meat analysis [2,3], a comprehensive comparative evaluation of the performance of SVMs, ANNs, and Random Forests specifically for Raman spectroscopic identification of pure and mixed minced meat has been limited.

This study addresses this gap by evaluating the performance of SVM, ANN, and Random Forest models for the classification of pure and mixed minced meat samples. A particular focus is placed on the crucial influence of sample homogenization on spectral quality and subsequent model performance. The findings of this research will provide a detailed comparative analysis of these advanced machine learning approaches, contributing to the development of robust, rapid, and accurate tools for ensuring meat product authenticity and combating food fraud.

## 2. Materials and Methods

**Figure 1:**
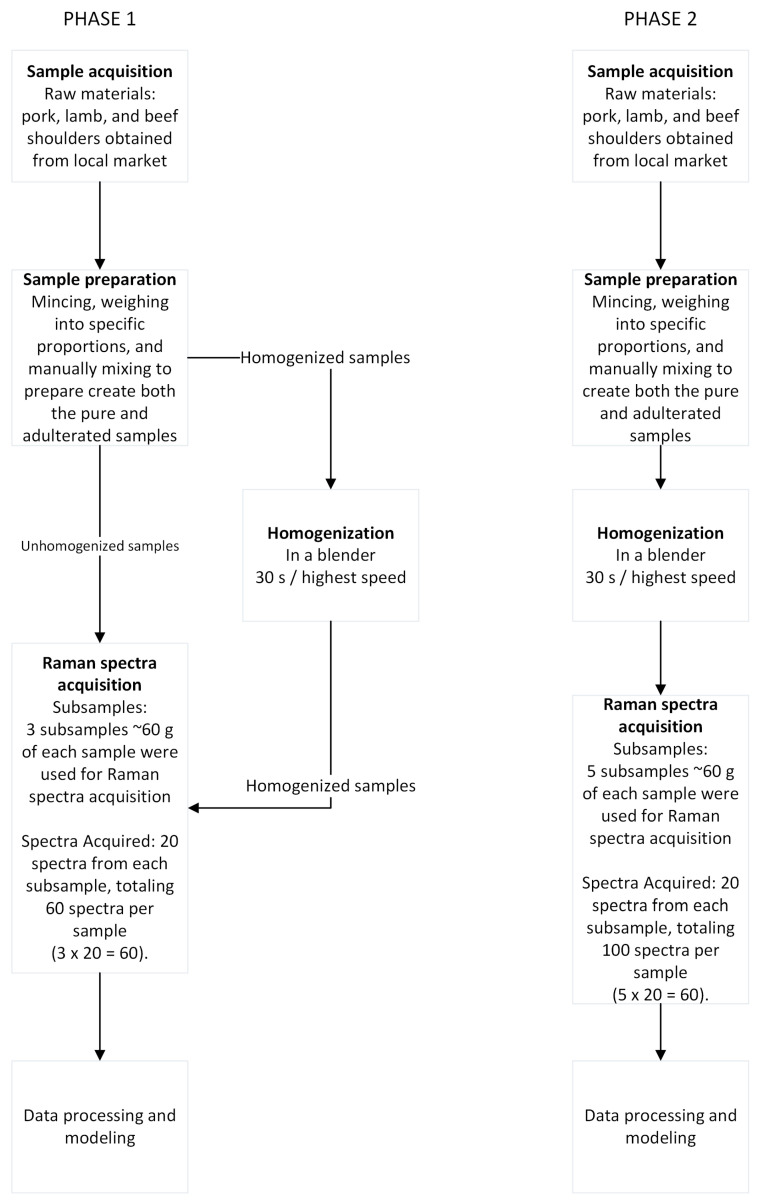
Process flow diagram of the experiment.

The overall experimental design, including sample preparation and data acquisition, is summarized in the process flow diagram (Figure 1).

### 2.1. Sample Preparation

For the minced meat samples, pure raw pork (*Sus scrofa domesticus*), lamb (*Ovis aries*), and beef (*Bos taurus*) shoulder were used. Raw meat was acquired from a local supermarket in chunks and immediately transported to the laboratory for sample preparation. Pure meat was minced in-house using a Bosch Meat Mincer ProPower 2000 W MFW67440 with a 3 mm plate. Pure minced meat was then used for mixture preparation at specific ratios, with the desired components mixed manually to ensure a homogeneous mixture. The study involved two distinct phases of sample preparation and data acquisition. Phase 1 (Figure 1) focused on the initial evaluation of homogenization impact and included five unhomogenized samples and five new homogenized samples of the same formulations: 100% pure minced pork shoulder (200 g)—P, 100% pure minced lamb shoulder (200 g)—L, 100% pure minced beef shoulder (200 g)—B, a 50%/50% pork/beef mixture (100 g pork + 100 g beef)—BP50, and a 50%/50% pork/lamb mixture (100 g pork + 100 g lamb)—LP50. For this phase, a total of 10 replicates were prepared (5 unhomogenized and 5 homogenized). From each physical sample, three subsamples of approximately 60 g were used for Raman spectra acquisition. A total of 20 spectra were acquired from each subsample, totaling 60 spectra per sample, resulting in a dataset of 600 spectra for this phase. The homogenized samples were prepared using a Bosch VitaBoost 6 1600 W (MMBH6P6B) blender, with each sample blended for 30 s at the highest speed to ensure uniform distribution and reduce spectral variability. Phase 2 (Figure 1) involved a more in-depth analysis of homogenized mixtures. The formulations used for this phase (9 in total) were as follows: 100% pure pork (300 g)—P, 100% pure lamb (300 g)—L, 100% pure beef (300 g)—B, a 25%/75% pork/beef mixture (75 g pork + 225 g beef)—BP75, a 50%/50% pork/beef mixture (150 g pork + 150 g beef)—BP50, a 75%/25% pork/beef mixture (225 g pork + 75 g beef)—BP25, a 25%/75% pork/lamb mixture (75 g pork + 225 g lamb)—LP75, a 50%/50% pork/lamb mixture (150 g pork + 150 g lamb)—LP50, and a 75%/25% pork/lamb mixture (225 g pork + 75 g lamb)—LP25. For each of these formulations, five subsamples of approximately 60 g were used for Raman spectra acquisition. A total of 20 spectra were acquired from each subsample, totaling 100 spectra per sample, to create a larger dataset for this phase. Homogenization for these samples was performed identically to Phase 1 using the Bosch VitaBoost 6 1600 W (MMBH6P6B) blender for 30 s at the highest speed. Each subsample was placed in a disposable dish (1.5 cm deep) prior to data collection.

### 2.2. Raman Spectroscopy Acquisition

Raman spectra were acquired using a Horiba Jobin Yvon Xplora instrument (Horiba Jobin Yvon S.A.S., Villeneuve-d’Ascq, France) equipped with a BX51 microscope (Olympus, Tokyo, Japan). The key acquisition parameters were consistent with our previous work in this field: objective lens: 10× (numerical aperture: 0.25), grating: 1200 lines/mm, exposure time: 20 s (achieved by combining two 10 s acquisitions to facilitate cosmic spike removal), laser wavelength: 785 nm (at 90 mW power), detector: thermoelectrically cooled CCD (Syncerity, Horiba Scientific, Edison, NJ, USA) operating at −60 °C, and spectral range: 200–3200 cm^−1^. LabSpec 6 software (Horiba Scientific, Villeneuve-d’Ascq, France) was used for spectral acquisition. Our choice of a 785 nm laser is a common practice in food science applications, as it reduces fluorescence interference that can occur at shorter wavelengths, as observed in studies by Boyaci et al. [10] and Liu et al. [13]. The laser power (90 mW) and exposure time (20 s) were carefully chosen as a balance between acquiring a strong signal and avoiding sample degradation, which is in line with the power settings (90–100 mW) and similar acquisition times (15–30 s) used by other researchers on similar samples [9,10,14]. Samples were stored at 1 °C and then allowed to equilibrate to room temperature for 10 min before spectral acquisition.

### 2.3. Data Analysis and Machine Learning

All data processing, statistical analysis, and the development of machine learning models were conducted using the R programming language (version 4.4.0) [15] and various specialized R packages. The primary aim was to build predictive models that can classify different meat types and detect adulteration based on their Raman spectra. Key R packages utilized include hyperSpec (v0.100.2) [16] for spectroscopic data handling; MASS (v7.3.60.2) [17], caret (v7.0.1) [18], e1071 (v1.7.16) [19], nnet (v7.3.19) [17], and randomForest (v4.7.1.2) [20] for machine learning; ggplot2 (v3.5.2) [21] for visualization; and Peaks (v0.2) [22] for signal processing. The analytical workflow for developing these machine learning models involved several steps. The analysis consisted of three parts: preprocessing of the Raman spectra, exploratory analysis, and model development (including calibration, validation, and testing). First, for data preparation and preprocessing, the raw Raman spectral data, encompassing measurements from both pure meat samples and various mixtures, underwent several steps, including cosmic spike removal, spectral range selection (from 600 to 1800 cm^−1^), baseline correction, and normalization. Baseline correction was performed using an iterative algorithm to mathematically estimate and remove the broad background fluorescence and instrumental noise from the spectra. Spectra were then normalized by dividing each data point by the spectrum’s mean intensity to ensure comparable scales across all samples. These preprocessing techniques were consistent with those employed in our previous work on Raman data from similar food matrices [11,12,23]. This process was performed to minimize instrumental noise, remove background fluorescence, and ensure spectral features were comparable across all samples. Second, for exploratory analysis, Principal Component Analysis (PCA) was employed to gain insight into the high-dimensional spectral datasets and visualize potential clustering of different meat types. Additionally, loadings were used to interpret the spectral information contained in each principal component. Third, for machine learning model development and evaluation, the preprocessed data was partitioned into a training set and an independent testing set to ensure the model’s performance could be reliably assessed on unseen data. The initial data split into training and testing sets was performed randomly; however, we confirmed that the class proportions remained relatively consistent and maintained the overall class ratio. Each model’s configuration was specifically tuned on a training data set for this classification task. This process involved optimizing key hyperparameters through a grid search method. The following hyperparameters were tuned for each model: SVM’s cost (C) parameter, ANN’s number of neurons and weight decay, and the optimal number of variables to randomly sample at each split for the Random Forest model. The best-performing model parameters were used for the final analysis, as determined by leave-one-out cross-validation (SVM and Random Forest) or 10-fold cross-validation (ANN). For SVM, a linear kernel was used with an optimized cost (C) parameter of 1. To find the most discriminative wavelengths from a Support Vector Machine (SVM) model, we examined the model coefficients. ANN was configured as a single-hidden-layer network with 15 neurons using a sigmoid activation function for the hidden layer. The model was trained using a backpropagation algorithm with an optimized learning rate and a maximum of 20,000 epochs. Early stopping with a patience of 50 was implemented as a regularization technique to prevent overfitting. The optimal number of variables to randomly sample at each split for the RF model was determined to be 20 through a grid search. Following this, the optimal number of trees was determined by analyzing the out-of-bag error rate plot to ensure stability and prevent overfitting. Additionally, the variable importance measure from the RF model was used to identify the most significant wavelengths for classification. Fourth, for performance assessment, all the developed models were evaluated using the independent testing set. Standard statistical measures, such as accuracy (the proportion of correctly classified samples) and the Kappa coefficient (a measure of agreement between predicted and actual classes, accounting for chance agreement), were calculated. Additionally, detailed metrics, including precision, recall, and F1-score, were generated to provide a comprehensive breakdown of correct and incorrect classifications for each meat type.

## 3. Results

### 3.1. Raman Spectra

**Figure 2:**
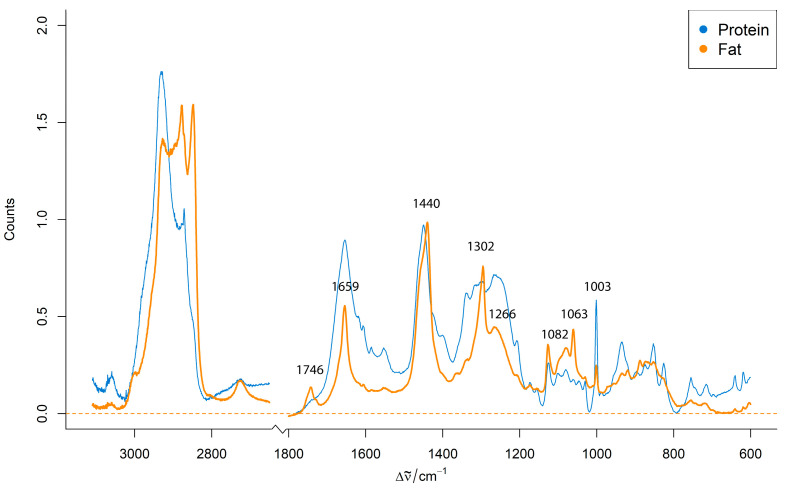
Representative preprocessed Raman spectra from protein-rich and fat-rich regions of an unhomogenized pork minced meat sample.

Figure 2 shows representative preprocessed Raman spectra obtained from distinct protein-rich and fat-rich regions within an unhomogenized pork minced meat sample from the first phase of the experiment.

The spectra display characteristic bands associated with major biochemical components of meat, primarily lipids and proteins. The characteristic Raman bands of minced meat are summarized in Table 1. The representative preprocessed Raman spectra from protein-rich and fat-rich regions within an unhomogenized pork minced meat sample (Figure 2) display these characteristic bands, highlighting the primary biochemical components of meat. The presence of these distinct spectral signatures for protein and fat within the same sample highlights the inherent compositional heterogeneity of raw minced meat. This variability presents a significant challenge for direct, consistent spectroscopic analysis and underscores the necessity for appropriate sample preparation techniques to achieve reliable and discriminative Raman signals across different meat species.

**Figure 3:**
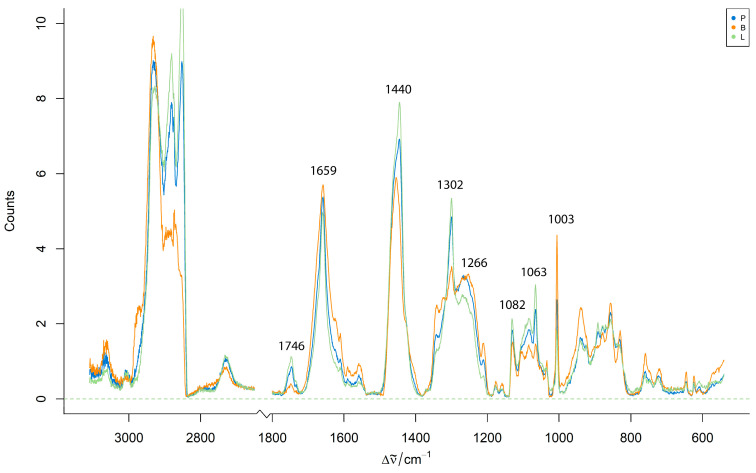
Mean preprocessed Raman spectra from homogenized pure pork, lamb, and beef minced meat samples.

For a comparative analysis of the species, Figure 3 shows the mean preprocessed Raman spectra from homogenized pure pork, lamb, and beef samples. These spectra show visible differences in the intensity of the key Raman bands, particularly around the 1659 cm^−1^ (C=C stretching) and 1440 cm^−1^ (C–H bending) regions.

### 3.2. Exploratory Analysis

**Figure 4:**
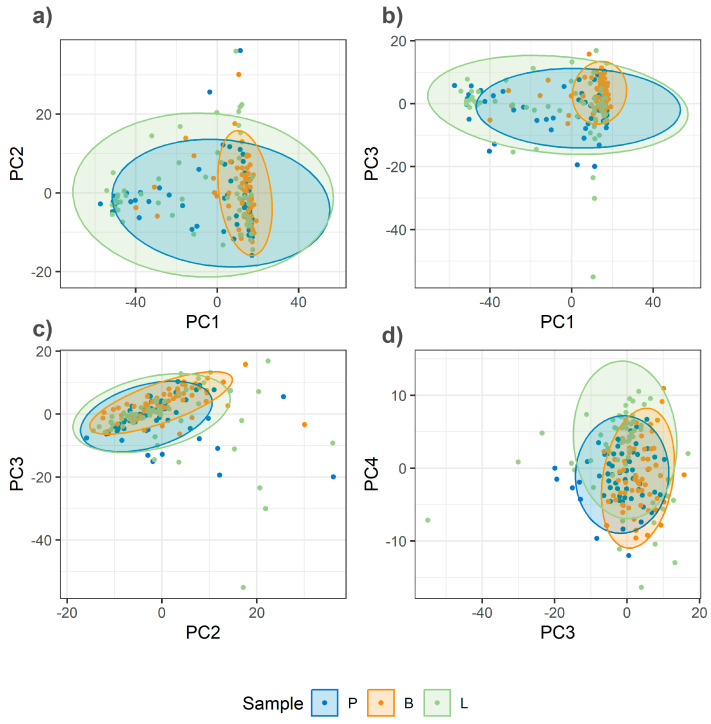
PCA score plots of Raman spectra from unhomogenized minced meat samples (pork, lamb, and beef). (**a**) PC1 vs. PC2; (**b**) PC1 vs. PC3; (**c**) PC2 vs. PC3; (**d**) PC3 vs. PC4.

**Figure 5:**
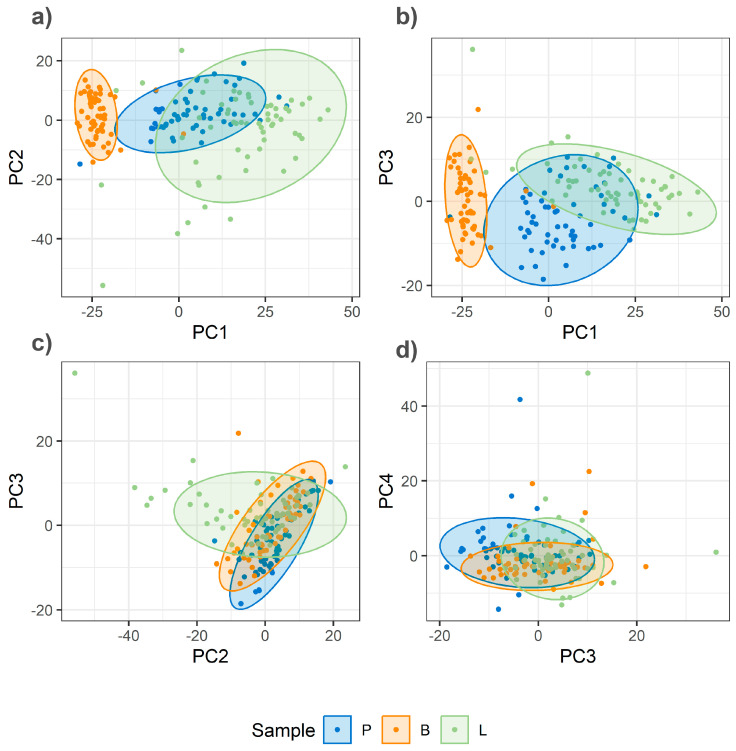
PCA score plots of Raman spectra from homogenized minced meat samples (pork, lamb, and beef). (**a**) PC1 vs. PC2; (**b**) PC1 vs. PC3; (**c**) PC2 vs. PC3; (**d**) PC3 vs. PC4.

The critical impact of homogenization on spectral quality and class separation is demonstrated by a comparative analysis of PCA score plots for unhomogenized and homogenized samples (Figure 4 and Figure 5). The PCA of unhomogenized samples reveals a high degree of overlap among the data points for different meat species (Figure 4). The percentage of variance explained by the first four principal components is as follows: PC1—23%, PC2—9%, PC3—8%, and PC4—5%.

**Figure 6:**
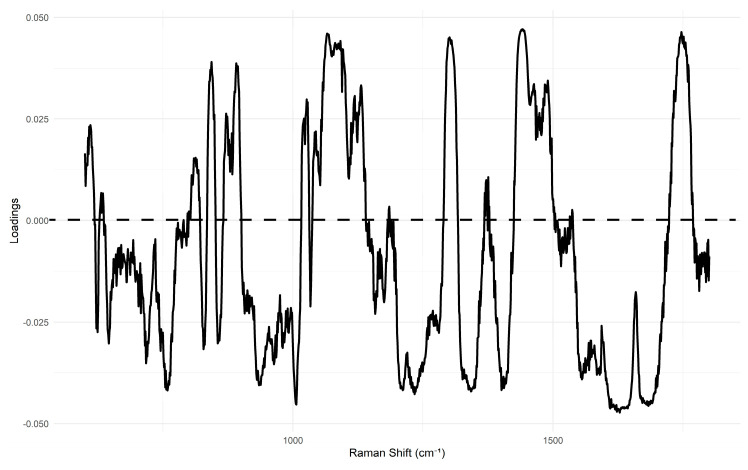
PCA loading plot of PC1 of Raman spectra from homogenized minced meat samples (pork, lamb, and beef).

In contrast, the PCA score plot for homogenized samples (Figure 5) demonstrates a visibly improved separation, particularly along the lines of PC1. In this PCA, the first four principal components explain the total variance as follows: PC1—20%, PC2—10%, PC3—7%, and PC4—7%. The analysis of the loadings for these principal components, particularly PC1, reveals high contributions from bands at 1659 cm^−1^ and 1440 cm^−1^ (Figure 6), among others. This suggests that PC1 primarily captures differences related to fat composition and, more specifically, the level of unsaturation, as the intensity ratio of these bands is shown to be an indicator of this property (insert citation).

### 3.3. Machine Learning Classification Performance

The classification performance of the SVM, ANN, and Random Forest models on various minced meat samples is summarized in Table 2. The data implies that homogenization dramatically improves performance across all algorithms and sample compositions.

For pure meat samples, all models achieve high accuracies after homogenization, confirming that this preprocessing step is sufficient to enable robust classification. SVM and ANN consistently demonstrate the highest accuracies, reaching 0.95 and 0.93, respectively, with corresponding high Kappa values. This indicates that these models are highly effective at capturing the biochemical differences between species.

The analysis of complex mixtures with varying adulteration ratios presents a more nuanced challenge. While all models show reduced performance with the introduction of mixtures, a clear hierarchy of performance can be observed. SVM continues to deliver the highest accuracy for the pork/lamb mixtures (0.86), demonstrating its robustness in classifying samples with a wider range of concentrations. In contrast, all models struggle with the pork/beef mixtures, with accuracies dropping significantly (e.g., SVM at 0.56). This suggests that the spectral similarity between pork and beef is a greater challenge for these algorithms than the spectral differences between pork and lamb.

The most influential wavelengths for the SVM model vary depending on the classification task. For the homogenized pure meat samples (pork, lamb, and beef) from the first phase, the top three influential wavelengths are around the bands 1740, 1045, and 1202 cm^−1^, with corresponding coefficient values of 0.0336, 0.0317, and 0.0310. For the more complex pork/lamb mixture model, the most influential wavelengths are a combination of bands including 853, 1138, and 1655 cm^−1^, all with high coefficients (around 0.11–0.12). While these bands do not directly correspond to the most prominent peaks in the raw spectra, this finding highlights a known characteristic of SVMs in high-dimensional data. Rather than relying on single, obvious peaks, the model may be leveraging subtle spectral features, noise, or complex interactions between correlated wavelengths to optimize its classification performance. In contrast, the model for the challenging pork/beef mixtures relies heavily on a narrow spectral region, with the top influential wavelengths all clustered in the 1230–1260 cm^−1^ range (amide III). This reliance on a specific protein-related band, along with the lower model performance, highlights the significant spectral similarity between these two species, making them a more difficult classification challenge.

### 3.4. Specific Confusion Matrices (Example)

Table 3 presents the confusion matrix and associated statistics for the Support Vector Machine (SVM) model’s performance on the comprehensive homogenized pork/lamb (P, L, LP25, LP50, and LP75) mixtures. This model achieves a high overall accuracy of 0.86 and a Kappa coefficient of 0.82.

The detailed, per-class metrics reveal the model’s strengths and weaknesses within this multi-class scenario. The model demonstrates excellent performance in identifying the pure samples, achieving a perfect recall of 1.00 for pure pork and a high recall of 0.94 for pure lamb. This indicates that the model is highly effective at correctly identifying the pure samples from all other classes.

Performance for the mixed classes is also robust but with some nuanced challenges. While the model achieves a good F1-score for the LP25, LP50, and LP75 classes (0.81, 0.78, and 0.87, respectively), the matrix shows that misclassifications often occur between adjacent concentration percentages. For instance, some true LP25 samples are incorrectly classified as LP50, and some true LP50 samples are classified as LP25. This suggests that while the model successfully differentiates the mixed classes from the pure classes, the subtle spectral differences between very similar mixture ratios present a more difficult classification challenge.

Table 4 presents the confusion matrix and associated statistics for the SVM model’s performance on the comprehensive homogenized pork/beef (P, B, BP25, BP50, and BP75) mixtures. This model achieves an overall accuracy of 0.56 and a Kappa coefficient of 0.45.

The detailed per-class metrics reveal the significant challenges in distinguishing between pork and beef, particularly in mixtures. While the model shows relatively good performance on the pure classes (e.g., a precision of 0.79 for pure pork and a recall of 0.54 for pure beef), its performance drops considerably for the mixed samples. The lowest precision and F1-scores are observed for the mixed classes, especially BP50 (F1-score of 0.54) and BP75 (F1-score of 0.37). This indicates that the model struggles to reliably identify these specific mixture ratios. The high degree of misclassification between adjacent mixed classes and with the pure classes (e.g., true BP75 samples being classified as BP25) highlights the high spectral similarity between pork and beef. This confusion is a direct result of the models’ difficulty in discerning subtle changes in chemical composition when the meat from these two species is mixed.

## 4. Discussion

The findings of this study suggest that homogenization can be a critical pre-analytical step for achieving high classification accuracy with spectroscopic methods. As seen in the PCA score plots for non-homogenized samples, the data points were scattered, indicating high spectral variability within each meat group. Conversely, homogenized samples showed more distinct clusters, which consequently contributed to the improved performance of all classification models. This finding reinforces the need to standardize sample preparation for reliable spectroscopic analysis. The high classification accuracies we obtained are in strong agreement with the broader literature on food authenticity [4,5,6]. These studies emphasize that spectroscopic technologies are increasingly recognized as powerful, metabolite-based detection methods for meat adulteration. They are valued as rapid, nondestructive, and cost-effective alternatives to more traditional techniques like ELISA and Mass Spectrometry, which often require extensive sample preparation and are time-consuming [4,7].

The use of AI models to classify spectroscopic data is also well established for meat authentication. Our results show that all three machine learning models—ANN, RF, and SVM—performed well in discriminating pure meat species. This aligns with other studies, such as the work by Fengou et al. (2021) [3], where Support Vector Machines (SVMs) with spectroscopy-based sensors were successfully employed to detect adulteration in minced meat. Moreover, the successful use of Raman spectroscopy to classify beef based on its production system has been independently validated by other research. Logan et al. (2022) [14], for example, used Raman spectroscopy and Partial Least Squares Discriminant Analysis (PLS-DA) to accurately classify beef from different Australian production regions, achieving classification accuracies of up to 99%. These results, along with our own, provide strong evidence that Raman spectroscopy, when combined with appropriate chemometric models, is a highly effective tool for meat authentication.

In order to provide a more in-depth analysis with chemical interpretation, we used PCA and SVM coefficients to identify the most influential wavelengths for our classifications. The PCA analysis was critical for understanding the chemical basis of our classifications. The lower percentage of variance explained by the first few principal components (PCs) in our PCA models, for both unhomogenized and homogenized samples, highlights the high dimensionality and complexity of Raman spectral data from food matrices. This reinforces the general reputation of Raman spectra as a specific molecular fingerprint that cannot be easily simplified into a few dominant components. The PCA loadings, particularly for PC1, revealed high contributions from bands at approximately 1659 cm^−1^ (C=C stretching) and 1440 cm^−1^ (C–H bending). This suggests that PC1 primarily captures differences related to fat composition and the level of unsaturation, as the intensity ratio of these bands is reported to be an indicator of this property [25].

The analysis of the SVM coefficients provides an intriguing insight into the model’s decision-making process. The most influential wavelengths for classification were found to be at 1739 cm^−1^, 1045 cm^−1^, and 1202.31 cm^−1^. While these bands are not the most prominent or frequently discussed peaks in the literature, this outcome may be attributed to several factors. First, in high-dimensional data, SVMs can assign importance to subtle features that may not be visually apparent, often capitalizing on combinations or ratios of correlated wavelengths rather than on individual, isolated peaks. Second, given the complexity of the minced meat matrix, the model may be detecting minor spectral differences in key components such as lipids, collagen, and myofibrillar proteins, whose peaks may be obscured but are still highly discriminative. This highlights a key strength of machine learning models in their ability to uncover complex patterns that are not easily interpretable by visual inspection alone. This finding contrasts with the PCA loadings, which showed high contributions from the more well-known 1659 cm^−1^ and 1440 cm^−1^ bands, and suggests that different models may prioritize different spectral information to achieve their respective performance outcomes.

Despite the promising results, this study has certain limitations. The random partitioning of spectra into training and testing sets carries a potential risk of data leakage, as spectra from the same physical sample could appear in both subsets, which may lead to an overestimation of model performance. Additionally, our study focused on specific pure meat types and their binary mixtures from a single batch of samples; future work could include a wider range of animal species, different cuts of meat, and the impact of other processing parameters (e.g., freezing–thawing cycles, different fat content levels), which can influence spectral signatures. To address the issue of the challenging pork/beef mixtures and improve accuracy, future work could explore several technical solutions. This could include focusing on specific spectral ranges that are more indicative of compositional differences, such as the lipid region (1650–1670 cm^−1^); using advanced variable selection methods; or integrating complementary techniques like Fourier-transform infrared spectroscopy (FT-IR) to provide a more comprehensive chemical fingerprint.

For potential industrial implementation, a comprehensive calibration process is essential. This requires building a robust calibration set with spectra from a wide range of real-world samples, including different breeds, production systems, and fat content levels. Such extensive calibration would ensure the model’s robustness and generalizability, enabling its integration into a portable, on-site Raman device for rapid and cost-effective quality control.

## 5. Conclusions

This study demonstrates the applicability of Raman spectroscopy combined with machine learning for the accurate classification of pure and mixed minced meat samples. The research highlights the critical role of sample homogenization as a pre-analytical step for improving the quality of Raman spectral data and, consequently, enhancing the performance of classification models for this complex food matrix. While all evaluated algorithms showed promise, SVM generally delivered the highest classification accuracy for the investigated meat mixtures, particularly for pure species and pork/lamb combinations.

In contrast to the success of our classification models, this work encountered a challenge in interpreting the underlying chemical mechanisms that drive their decisions. This limitation, combined with the inherent challenges of the analyzed food matrix and the limitations of our study, highlights the power of machine learning techniques. Our findings show that these models can be powerful predictive tools, even when the specific spectral features they rely on are not easily interpretable by traditional methods.

Despite the promising results, this study has certain shortcomings, such as the use of a single batch of samples and the potential risk of data leakage from the random data-splitting strategy. To address these limitations, future work should focus on a wider range of animal species, different cuts of meat, and a more rigorous data-splitting methodology. This would improve the robustness and generalizability of the models. If these obstacles can be tackled, this rapid and effective analytical approach has the potential to be integrated into routine industrial settings. However, for full industrial implementation, a comprehensive calibration process is essential. This requires building a robust calibration set from a wide range of real-world samples to ensure the model’s generalizability. Such a model could then be integrated into a portable, on-site Raman device for rapid, non-destructive, and cost-effective quality control.

## Figures and Tables

**Table 1:** Characteristic Raman bands for key biochemical components of minced meat [9,23,24].

Raman Shift (cm^−1^)	Assignment	Associated Component
1746	C=O stretching vibration from triglycerides	Fat
1659	C=C cis double bond stretching vibration, characteristic of unsaturated fatty acids	Fat
1440	C–H bending from methylene groups	Fat
1302	C–H twisting vibration of the –CH_2_ group	Fat
1266	C–H bending vibration at the cis double bond in R–HC=(H–R)	Fat
1082 and 1063	C–C stretching vibration	Fat
969	C–N asymmetric stretching of the choline group (CH_3_)_3_N^+^	Fat
1650–1680	Amide I	Protein
1230–1300	Amide III	Protein
1003	Phenylalanine ring breathing mode	Protein

**Table 2:** Performance of machine learning algorithms for classifying minced meat samples based on Raman spectra.

Treatment	Samples Spectra Used in Model	Machine Learning Algorithm	Accuracy of Classification Model	Kappa
Unhomogenized	P, B, L	SVM	0.72	0.584
		ANN	0.47	0.209
		Random Forests	0.62	0.439
	P, BP50, B	SVM	0.61	0.099
		ANN	0.66	0.487
		Random Forests	0.51	0.268
	P, LP50, L	SVM	0.62	0.419
		ANN	0.55	0.316
		Random Forests	0.55	0.326
Homogenized	P, B, L	SVM	0.95	0.922
		ANN	0.93	0.896
		Random Forests	0.86	0.793
	P, BP50, B	SVM	0.88	0.820
		ANN	0.85	0.096
		Random Forests	0.86	0.262
	P, LP50, L	SVM	0.68	0.518
		ANN	0.65	0.473
		Random Forests	0.65	0.473
Homogenized	P, B, L	SVM	0.88	0.820
		ANN	0.77	0.112
		Random Forests	0.71	0.113
	P, BP25, BP50, BP75, B	SVM	0.56	0.454
		ANN	0.51	0.386
		Random Forests	0.52	0.401
	P, LP25, LP50, LP75, L	SVM	0.86	0.820
		ANN	0.78	0.724
		Random Forests	0.69	0.612

**Table 3:** Confusion matrix and statistics for SVM classification of homogenized pork/lamb mixtures (P, L, LP25, LP50, and LP75).

**Sample**	**P**	**L**	**LP25**	**LP50**	**LP75**
P	30	0	0	0	0
L	0	34	1	1	1
LP25	2	1	30	1	4
LP50	7	1	1	20	0
LP75	0	0	4	0	29
Additional metrics					
Precision	1.00	0.92	0.79	0.69	0.88
Recall	0.77	0.94	0.83	0.91	0.86
F1	0.87	0.93	0.81	0.78	0.87
Overall Statistics: Accuracy: 0.86, Kappa: 0.82					

**Table 4:** Confusion matrix and statistics for SVM classification of homogenized pork/beef mixtures (P, B, BP25, BP50, and BP75).

**Sample**	**P**	**B**	**BP25**	**BP50**	**BP75**
P	27	1	0	5	1
B	1	19	1	11	1
BP25	0	7	20	0	4
BP50	5	4	4	15	5
BP75	6	5	11	1	13
Additional metrics					
Precision	0.79	0.58	0.65	0.45	0.36
Recall	0.69	0.53	0.56	0.68	0.38
F1	0.74	0.55	0.60	0.55	0.37
Overall Statistics: Accuracy: 0.5629, Kappa: 0.4535					

## Data Availability

The data presented in this study are available on request from the corresponding author. (The data are not publicly available due to privacy or ethical restrictions.)

## Abbreviations

The following abbreviations are used in this manuscript:

ANN	Artificial Neural Network
RF	Random Forest
SVM	Support Vector Machine
